# Clinical relevance of molecular identification of microorganisms and detection of antimicrobial resistance genes in bloodstream infections of paediatric cancer patients

**DOI:** 10.1186/s12879-016-1792-8

**Published:** 2016-09-01

**Authors:** Fabianne Carlesse, Paola Cappellano, Milene Gonçalves Quiles, Liana Carballo Menezes, Antonio Sérgio Petrilli, Antonio Carlos Pignatari

**Affiliations:** 1 Institute of Paediatric Oncology, Universidade Federal de São Paulo, Rua Botucatu 743, São Paulo, 04037020 Brazil; 2 Infectious Diseases Division, Universidade Federal de São Paulo, São Paulo, Brazil

## Abstract

**Background:**

Bloodstream infections (BSIs) are the major cause of mortality in cancer patients. Molecular techniques are used for rapid diagnosis of BSI, allowing early therapy and improving survival. We aimed to establish whether real-time quantitative polymerase chain reaction (qPCR) could improve early diagnosis and therapy in paediatric cancer patients, and describe the predominant pathogens of BSI and their antimicrobial susceptibility.

**Methods:**

Blood samples were processed by the BACTEC system and microbial identification and susceptibility tests were performed by the Phoenix system. All samples were screened by multiplex 16 s rDNA qPCR. Seventeen species were evaluated using sex-specific TaqMan probes and resistance genes *bla*SHV, *bla*TEM, *bla*CTX, *bla*KPC, *bla*IMP, *bla*SPM, *bla*VIM, *van*A, *van*B and *mec*A were screened by SYBR Green reactions. Therapeutic efficacy was evaluated at the time of positive blood culture and at final phenotypic identification and antimicrobial susceptibility results.

**Results:**

We analyzed 69 episodes of BSI from 64 patients. Gram-positive bacteria were identified in 61 % of the samples, Gram-negative bacteria in 32 % and fungi in 7 %. There was 78.2 % of agreement between the phenotypic and molecular methods in final species identification. The *mec*A gene was detected in 81.4 % of *Staphylococcus* spp., and 91.6 % were concordant with the phenotypic method. Detection of *van*A gene was 100 % concordant. The concordance for Gram-negative susceptibilities was 71.4 % for Enterobacteriaceae and 50 % for *Pseudomonas aeruginosa*. Therapy was more frequently inadequate in patients who died, and the molecular test was concordant with the phenotypic susceptibility test in 50 %.

**Conclusions:**

qPCR has potential indication for early identification of pathogens and antimicrobial resistance genes from BSI in paediatric cancer patients and may improve antimicrobial therapy.

## Background

Chemotherapy for childhood cancer is one of the greatest successes of modern medicine and the overall survival is now over 70 %. However myelossupression is a consequence of virtually all chemotherapy regimens and profound neutropenia (<0,5 × 10^9^) is a major risk factor for overwhelming infections [1].

The relative frequency of different organisms causing bacterial infection may vary with time period and geographical location. Regardless of these variations in causative organism, and the widespread use and availability of powerful antibiotics, bacteremia/sepsis remains the most important independent prognostic marker for mortality in children with cancer who have febrile neutropenia. A diagnosis of sepsis or bacteremia increased in 10-fold increase risk of death [2].

In 40 % of children that have clinical diagnosis of sepsis and 75 % of those with high-risk FN, the microorganisms cannot be detected by currently available conventional microbiological techniques [3]. Early empirical treatment of fever in cancer patients remains the standard of care for the management of febrile neutropenia (FN).

In this context, our group developed an in-house, real-time, quantitative polymerase chain reaction (qPCR) directly from BACTEC blood culture (BC) bottles to identify the main pathogens and important resistance genes causing bloodstream infection (BSI) [4]. The aim of this study was to evaluate if this technique could anticipate and improve the diagnosis and therapy in paediatric cancer patients. The secondary objective was to describe the main pathogens of BSI and their antimicrobial susceptibilities.

## Methods

### Study design

Prospective study between March 2011 and March 2012 at the Institute of Paediatric Oncology, São Paulo, Brazil. Informed consent was obtained from all patients and the study was approved by the Clinical Research Ethics Committee of the Federal University of São Paulo. The samples were collected as part of standard care from patients suspected of BSI in the hospital. BSI was defined as the isolation of a bacterial or fungal pathogen from at least one BC.

All episodes of BSI were sub-classified as single agent (Gram-positive bacteria, Gram-negative bacteria, or fungi) or polymicrobial if two or more pathogens were isolated from a single BC.

We included only the BSI episodes for which BACTEC bottles were available for molecular tests without selection. We excluded polymicrobial episodes because it is not feasible to compare the conventional method with the molecular method if more than one agent grows in the same BC.

### Data collection

Clinical data and bloodstream isolates were prospectively collected by local infection control practitioners.

### Definitions

Neutropenia was defined as an absolute neutrophil count of <500 cells/mm^3^ within 48 h after onset of bacteraemia [5]. Sepsis was defined as systemic inflammatory response syndrome in the presence of suspected or proven infection and organ dysfunction, according to international consensus guidelines. Septic shock was defined as sepsis with systolic blood pressure <90 mmHg that did not respond to adequate fluid resuscitation, and necessitated the use of a vasopressor. Severe sepsis was defined as inflammatory response syndrome triggered by a microorganism, identified or not, plus one of the following: cardiovascular organ dysfunction or acute respiratory distress syndrome; or dysfunction of two or more other organs: neurological, renal or hepatic [6].

Patients without any identifiable source of infection were classified as primary bacteraemia. Secondary BSIs were regarded as those with a clear source of bacteraemia other than a central line. Sources of secondary BSI were identified by culture of samples obtained from distant sites that yielded the same pathogen with an identical resistance pattern. Distant sites were sites other than a central line where an infection was diagnosed (e.g. pneumonia, urinary tract infection or abdominal infection). Intensive care unit (ICU) stay was defined as current stay in the ICU at onset of bacteraemia, regardless of previous history of ICU admission.

Empirical antimicrobial therapy was instituted according to the standard protocol of the institution [7] at the time of fever and suspicion of BSI after collection of blood culture. Antimicrobial therapy was analysed in two periods: Period 1: antibiotics in use when the Gram stain test of a positive bloodstream culture was available to the physician; and Period 2: antibiotics in use when the final results of microbial identification and antimicrobial susceptibility test were reported to the physician. Antimicrobial therapy was considered adequate if the treatment regimen included one or more antibiotics active in vitro against the microorganism identified by phenotypic tests.

Mortality associated with infection was defined as death within 14 days of bacteraemia and there was no other explanation for that.

### Microbiological features

BCs were performed using the BACTEC 9240 instrument (Becton & Dickinson, Microbiology Systems, Cockeysville, MD, USA). Identification at the species level and susceptibility testing were performed using the automated system Phoenix 100 (Becton & Dickinson Microbiology Systems). Minimum inhibitory concentration (MIC) determination was done by E test (AB Biodisk, Sweden) for oxacillin and carbapenems and by agar dilution for vancomycin, to confirm resistant phenotypes.

### Molecular detection of microorganisms and resistance genes

Total DNA was extracted from 500 μL of positive or negative BC broth after incubation on BACTEC 9240, using the phenol–chloroform method (Brazol; LGC Biotechnologia, Cotia, Brazil) with 300-mg glass beads (0.3-mm diameter; Scientific Industries, Bohemia, NY, USA) and processed in a disruptor Genie (Scientific Industries) for 10 min to achieve cell lysis [4].

Real-time qPCR was performed as follows according to the protocols published by Menezes et al. in 2013 [4, 8]. Taqman-based multiplex detection of 16S rDNA gene with Gram-positive- and Gram-negative-specific probes; single SYBR green detection of each following resistance genes *bla*_SHV_, *bla*_TEM_, *bla*_CTX-M_, *bla*_IMP_, *bla*_SPM_, *bla*_GIM,_*bla*_SIM_*bla*_VIM_, *bla*_KPC_, *van*A, *van*B and *mec*A; and single Taqman-based detection of *Enterococcus* spp., coagulase-negative *Staphylococcus* (CoNS), *Staphylococcus aureus*, *Streptococcus pneumoniae*, *Escherichia coli*, *Klebsiella pneumoniae*, *Enterobacter cloacae*, *Proteus mirabilis*, *Salmonella* spp., *Serratia marcescens*, *Acinetobacter baumannii*, *Pseudomonas aeruginosa*, *Stenotrophomonas maltophilia*, *Mycobacterium tuberculosis*, *Aspergillus* spp., *Candida* spp. and *Fusarium* spp. were performed on an ABI 7500 real-time PCR System (Applied Biosystems, Foster City, CA, USA) instrument and quantified online and at the endpoint with sequence detection system software (version 2.0; Applied Biosystems) [4]. The primers were checked for specificity in a BLAST search available through the NCBI website (http://blast.ncbi.nlm.nih.gov/Blast.cgi). A primer set for the haemochromatosis gene was designed as an internal control for DNA extraction.

Resistance gene amplification by SYBR Green monoplex real-time PCR was performed in a 25-μL reaction mixture containing 12.5 μL Platinum SYBR Green qPCR SuperMix (Invitrogen, Carlsbad, CA, USA), 0.5 μM each primer, and 5 μL purified DNA extracted from samples. The real-time PCR conditions were as follows: 50 °C for 2 min and 95 °C for 10 min; 40 cycles of 95 °C for 15 s and 60 °C for 60 s; and a melting curve step (from 68 to 95 °C in increments of 0.5 °C/s). The metallo-β-lactamase amplification was performed by SYBR-based multiplex real-time PCR.

The samples with discordant antimicrobial resistance results between molecular and phenotypic analyses were submitted to a qPCR assay directly from of the isolates strains. For methicillin-resistant *Staphylococcus* strains with discordant results, we determined the MIC for oxacillin with E test (fabricante) and the diameter zone for cefoxitin with the disc diffusion method following the Clinical and Laboratory Standards Institute recommendations.

Data collection was performed using SSPS version 17.0 software (SPSS Inc., Chicago, IL, USA) and Microsoft Office Excel 2010 (Microsoft, Redmond, WA, USA).

## Results

### Study population and patients characteristics

We identified 168 episodes of BSI, 81 episodes were included in the study with BACTEC BC bottles and were randomly available for molecular tests. Twelve episodes with polymicrobial culture results were excluded and our final data comprised 69 episodes of BSI from 64 patients.

Patients had a mean age of 7.8 years (range 0–22 years), and 57.8 % were male. The underlying diseases were: 20 % (13/64) acute lymphoid leukaemia; 19 % (12/64) central nervous system tumour; 14 % (9/64) acute myeloid leukaemia (AML); 9 % (6/64) non Hodgkin’s lymphoma; 8 % (5/64) neuroblastoma; 6 % (4/64) retinoblastoma; 5 % (3/64) osteosarcoma; 3 % (2/64) Ewing’s sarcoma; 2 % (1/64) Wilms’ tumour; and 20 % (13/64) others less common childhood tumours.

Among the potential factors predisposing to BSI, a central line catheter was present in 83 % (57/69), previous antimicrobial therapy was administered in 54 % (37/69), and neutropenia was present in 61 % (42/69). At onset of clinical evaluation, the use of antimicrobial therapy was observed in 85 % (59/69), sepsis was present in 68 % (47/69), septic shock occurred in 4 % (4/69), and severe sepsis in 3 % (2/69). Primary bacteraemia occurred in 91 % (63/69). Among secondary BSI caused by Gram-positive bacteria, the skin was the source in 60 %, followed by lungs and intestinal tract. In Gram-negative BSI, only one pulmonary secondary episode of BSI was identified.

Sixteen patients died during the study period, and six of these episodes occurred within 14 days and were related to BSI.

### Phenotypic bacterial identification

Among 69 BSI episodes, 61 % (42/69) were caused by Gram-positive bacteria, 32 % (22/69) by Gram-negative bacteria, and 7 % (5/69) by fungi (Table 1). The three major pathogens of BSI were: CoNS in 26 % (18/69), followed by *S. aureus* in 13 % (9/69), and *P. aeruginosa* in 12 % (8/69). Five episodes were caused by fungi: *Candida tropicalis*, *Candida parapsilosis*, *Candida guillermondi*, *Candida lusitaniae* and *Rhodotorula* spp. (Table 1).

**Table 1:** Rank order of bloodstream pathogens isolated from BSI in paediatric cancer patients

Agents	69 (100 %)
Gram positive	42 (61)
CoNS	18 (26)
*Staphylococcus aureus*	9 (13)
Viridans group *Streptococcus*	6 (9)
*Streptococcus* spp.	3 (4)
*Corynebacterium* spp.	2 (3)
*Enterococcus* spp.	2 (3)
Other Gram positive	2 (3)
Gram negative	22 (32)
*Pseudomonas. aeruginosa*	8 (12)
*Escherichia coli*	6 (9)
*Klebisiella pneumoniae*	3 (4)
*Enterobacter cloacae*	2 (3)
Other Gram negative	3 (4)
Fungi	5 (7)
*Candida tropicalis*	1 (1)
*Candida parapsilosis*	1 (1)
*Candida guillermondi*	1 (1)
*Candida lusitaniae*	1 (1)
*Rhodotorula* spp.	1 (1)

*CoNS* Coagulase negative *Staphylococci*

### Comparison of molecular with phenotypic identification methods

We performed phenotypic (automated) and molecular (qPCR) method for 69 positive samples taken directly from BC bottles and compared both methods. The overall concordance was 78.2 % for final species identification.

Among 42 samples positive for Gram-positive bacteria detected by BC processing, 36 were positive by Gram-postive 16 rDNA gene probe. Of these 36, 27 were identified at specie level by qPCR, 8 were identified by DNA sequencing and 7 were not identified. Three samples were discordant; in two of them the molecular method was not able to identify any microorganism, and one BC showed *S. aureus* and CoNS by qPCR. For 22 samples with Gram-negative isolates identified by BC processing, we observed a concordance of 81.8 % (18/22) with the molecular method. In two of these samples, qPCR was not able to identify the species, and in another two samples, the methods were discordant for species identification (Table 2).

**Table 2:** Discordant episodes between phenotypic and molecular tests of isolates from BSI in paediatric cancer patients

Episode	Phenotypic method	Molecular method
24	*Staphylococcus warneri*	Negative
29	*Staphylococcus aureus*	Negative
47	*Staphylococcus aureus*	CoNS
5	*Escherichia coli*	Negative
59	*Pseudomonas aeruginosa*	Negative
68	*Burkholderia cepacia*	CoNS/*Ralstonia pickettii*

*CoNS* Coagulase negative *Staphylococci*

Methicillin resistance was detected in 88 % (24/27) of the 27 *Sthaphylococcus* spp. samples identified by the phenotypic method. The *mec*A gene was detected in 22/24, corresponding to 91.6 % of concordance.

Two strains were identified as *Enterococcus* spp., and one of them was resistant to vancomycin by the phenotypic method. There was 100 % of concordance with detection of *vanA* by the molecular method.

The qPCR for extended spectrum β-lactamase (ESBL) coding genes was concordant with the phenotypic susceptibility profile test in 71.4 % (10/14) for Enterobacteriaceae.

Among four discordant samples, two were *E. coli* and had phenotypic detection of ESBL but no resistance gene was detected by the molecular method. One sample positive for *E. coli* with susceptibility to cephalosporins showed *bla*_CTX_, which was not detected directly from the isolated colony. The last discordant sample was positive for *E. cloacae*, with resistance to cephalosporins, but no resistance gene was detected. We did not detect any Enterobacteriaceae resistant to carbapenems by the phenotypic method and by the molecular method for *bla*_KPC_, *bla*_IMP_ and *bla*_VIM_ (Table 3).

**Table 3:** Resistance genes identified by molecular method and antimicrobial susceptibility for Gram-negative BSI

Episode	Phenotypic method	Resistance gene	Cefepime susceptibility	Meropenem susceptibility
1	*Klebsiella pneumoniae*	Negative	Susceptible	Susceptible
2	*Acinetobacter baumannii*	Negative	Susceptible	Susceptible
5	*Escherichia coli*	Negative	Resistant	Susceptible
6	*Escherichia coli*	Negative	Susceptible	Susceptible
8	*Serratia marcescens*	Negative	Susceptible	Susceptible
11	*Enterobacter cloacae*	Negative	Susceptible	Susceptible
16	*K. pneumoniae*	Negative	Susceptible	Susceptible
17	*Klebsiella oxytoca*	Negative	Susceptible	Susceptible
18	*Achromobacter* spp.	Negative	NT	NT
21	*Escherichia coli*	Negative	Susceptible	Susceptible
25	*Enterobacter cloacae*	Negative	Resistant	Susceptible
32	*Pseudomonas putida*	Negative	NT	NT
38	*Pseudomonas aeruginosa*	Negative	Resistant	Resistant
41	*Klebsiella pneumoniae*	*bla*_TEM_ *bla*_CTX_	Resistant	Susceptible
43	*Escherichia coli*	*bla*_CTX_	Susceptible	Susceptible
46	*Pseudomonas aeruginosa*	*bla*_SPM_	Resistant	Resistant
49	*Escherichia coli*	Negative	Resistant	Susceptible
50	*Pseudomonas aeruginosa*	Negative	Resistant	Susceptible
54	*Citrobacter freundii*	*bla*_CTX_	Resistant	Susceptible
59	*Pseudomonas aeruginosa*	Negative	Resistant	Resistant
65	*Escherichia coli*	Negative	Susceptible	Susceptible
68	*Burkholderia cepacia*	Negative	NT	Resistant

*NT* not tested

Eight samples were positive for non-fermenting Gram-negative bacteria by the phenotypic method, and qPCR for resistance genes was performed in seven, we excluded the *Achromobacter* spp. sample.

*Carbapenem resistance Pseudomonas aeruginosa* was phenotypically detected in four samples while two samples were negative for *bla*_SPM_ gene. There was 50 % concordance between both methods. One of these samples had the *bla*_SPM_ resistance gene detected when testing the isolate strain (Table 3).

Five samples were positive for fungi, four *Candida* spp. and one *Rhodotorula* spp., and the molecular method was able to identify 80 % of the samples with *Candida*.

### Antimicrobial therapy evaluation and death

Regarding antimicrobial therapy in Period 1, we observed inadequacy in 43 %. All except one episode were submitted to medical intervention at that time. In Period 2, the inadequacy remained in 12 % of episodes.

Death occurred in 16 % (11/69) of all the BSI episodes, and six of these episodes occurred within 14 days and were related to the BSI. The molecular method was concordant with phenotypic identification of the agent in 50 % of these episodes. Therapy in Period 1 was inadequate in patients whose death was related to BSI (5/6). In Period 2, three of them remained inadequate even after intervention. In these cases, we were able to identify *bla*_SPM_ in *P. aeruginosa* and *van*A in *Enterococcus faecium* by molecular test (Table 4).

**Table 4:** Phenotypic susceptibility, molecular tests and antimicrobial therapy of patients with death related to BSI

Episode	Underlying disease	Method		Phenotypic susceptibility			Resistance gene	Antimicrobial therapy	
		Phenotypic	Molecular	Cefepime	Meropenen	Vancomycin		Period 1	Period 2
5	AML	*E. coli*	Negative	Resistant	Susceptible	NA	Neg	Modified	Adequate
31	Other	*C. tropicalis*	*Candida* spp.	NA	NA	NA	NA	Adequate	Adequate
46	Wilms’ tumour	*P. aeruginosa*	*P. aeruginosa*	Resistant	Resistant	NA	*bla*_SPM_	Modified	Inadequate
56	ALL	*C. lusitanae*	Negative	NA	NA	NA	NA	Modified	Inadequate
58	ALL	*P. aeruginosa*	Negative	Resistant	Resistant	NA	Neg	Modified	Adequate
69	RMS	*E. faecium*	*Enterococcus* spp.	NA	NA	Resistant	*vanA*	Modified	Inadequate

*ALL* acute lymphoid leukaemia, *RMS* Rhabdomyosarcoma, *Neg* negative, *NA* not applicable

## Discussion

The strategy of using empirical antibiotic therapy has greatly influenced the outcome of fever in paediatric cancer patients. Unfortunately, BC can identify pathogens in only 40 % of children with fever and can take >24 h to yield a report [3].

We evaluated 69 episodes of BSI from 64 patients. The predominant underlying disease was haematological malignancy, this group of patients constitute the group of major risk for development of invasive bacterial infections, due to aggressive chemotherapy that results in long periods of neutropenia [9].

In our study, more than half of our patients were neutropenic, which has been recognised as an important risk factor for life-threatening infection since the 1960s when Bodey et al. identified a strong inverse correlation between the degree of neutropenia and the probability of infection [10–12].

We found a predominance of Gram-positive bacteria followed by Gram-negative bacteria, and fungi. Although we only studied a small sample, our results agree with our historic series and with the literature [7, 9, 13].

Among Gram-positive bacteria, CoNS was the main agent found, followed by *Staphylococcus aureus* which agrees with most epidemiological studies in this setting and is related with catheter implantation [14, 15].

Complications of *S. aureus* infection have been reported in paediatric cancer patients in 10–18 % of all infections and up to 33 % of cases of bacteraemia [16]. In our study, the majority of the patients with *S. aureus* infection had central venous catheter (CVC) (66 %) and primary bacteraemia. Mortality from *S. aureus* in paediatric oncologic setting is reported at 2.3 % [16]. No patient with *S. aureus* BSI died in our study.

Viridans group Streptococcus was the third in rank order of BSI in our patients. Infections due to viridans group *Streptococcus* have been seen especially in patients with AML, corresponding to ~25 % of all BSIs in these patients [17]. The mortality rate is high as 20 % [18]. In institutions with a high prevalence of this agent, the initial empirical antibiotic therapy includes vancomycin [19].

Among Gram-negative isolates, Enterobacteriaceae were predominant, with *E. coli* followed by *K. pneumoniae* as the main agents.

*Pseudomonas aeruginosa* was the main agent among non-fermenting bacteria and despite improvement in recent years, the prognosis of infection due this agent in this patient population remains poor, with case fatality rates exceeding 20 % [20]. Our institution has been treating patients with FN according to risk stratification for life-threatening infections; patients considered low risk receive moxifloxacin as outpatients, and high-risk patients receive cefepime as inpatients [7, 21].

We compared 69 positive BC episodes using phenotypic and molecular methods. The overall concordance was 78.2 % for final species identification, which is similar to a study conducted by Varani et al. in 2009 who found 79.2 % concordance between the two methods [22]. Comparing the two methods only for Gram-positive pathogens, the final species concordance identification was 89.6 %.

Recent studies have observed an increase in methicillin-resistant *Staphylococcus* infections, especially in patients with haematological malignancies; 70–80 % among CoNS and 25–30 % among *S. aureus* [23]. We detected 78 % methicillin resistance in BSI caused by *S. aureus*. The molecular method was able to detect the *mec*A gene in 93 % of these cases. Two samples were discordant, and when the molecular method was applied directly to the isolated colonies, it was concordant with the phenotypic method. Samples in which the phenotypic method shows methicillin resistance and the *mecA* gene is not found must be evaluated for *mecC*, and if this is present, patients must be managed as if they have methicillin-resistant *S. aureus* [24].

Resistance to vancomycin among enterococci has emerged as a general problem and is now found in patients with FN [25]. We found that 5 % of BSIs were due to *Enterococcus* spp., and 50 % were VRE. In our study we had one patient with VRE BSI infection who died 4 days after he had a positive BC and 1 day after the final result of BC emerged, which reflects the importance of early diagnosis. The molecular method was concordant in 100 % of the cases. Early identification of VRE is important beyond the therapeutic approach because this contributes to infection control procedures to avoid the spread of these microorganisms.

Three of our four *P. aeruginosa* isolates were resistant to carbapenems by the phenotypic method, which agreed with our molecular method in 50 % of cases. In the two discordant samples, we tested for the presence of the *MBL* genes directly from the isolated colonies; one of which was positive for *bla*_SPM_ and one negative for *MBL* genes. This suggests that another mechanism was responsible for carbapenem resistance, such as porin loss or efflux systems.

Even though the rate of infection with carbapenem-resistant *Acinetobacter* spp. is increasing, we found only one infection due to *A. baumannii* and it was susceptible to cefepime and carbapenems.

Enterobacteriaceae were predominant among Gram-negative isolates, and the main agent was *E. coli*, followed by *K. pneumoniae*, with a high concordance in final species identification between the two methods.

The literature shows an incidence of ESBL among *E. coli* varying from 12.6 to 48.3 % [26, 27]. In our study, 60 % of *E. coli* were ESBL producers, showing 78.5 % concordance with the molecular method. Three samples were discordant. Two of those had a phenotypic profile of ESBL and no resistance genes were detected. The other sample was susceptible to cephalosporins, and we found *bla*_CTX-M_, which was not confirmed in an additional test performed directly on the isolated colony. The patient received treatment with cefepime and had a good outcome.

Regarding fungal infection, our molecular method was able to identify yeasts, with the exception of one episode of *C. lusitaniae* that was responsible for one death. This is an uncommon species of *Candida* in these patients and it is important to note that it can be resistant to amphotericin B [28].

The impact of early appropriate antibiotic therapy on patient survival has been demonstrated in previous studies [1, 12, 29, 30]. In our study, we showed that only one of six patients who died within 14 days after BSI had adequate initial empirical therapy. Therapy was modified in five cases based on Gram staining of the positive BC (Period 1) and after the final results, three treatments remained inadequate. The molecular method was able to identify the microorganism and resistance gene in two of these three cases.

Early identification of the causative organism and resistance genes such as *vanA* for enterococci and *MBL* for *P. aeruginosa* is crucial for adequate antimicrobial therapy, especially in paediatric neutropenic patients. Our empirical treatment approach does not take into account the resistant microorganisms shown in our study.

A limitation of the method is that PCR is so sensitive that we could not exclude the risk of false positive from skin contamination during blood culture collection particularly for CoSN. Other molecular mechanisms of resistance could be added since our “in house” multiplex is done in an open qPCR plataform.

## Conclusions

The addition of molecular tests, including qPCR, for identification of pathogens and detection of antimicrobial resistance genes to the classical phenotypic tests in BSI is particularly relevant in paediatric cancer patients.

## Abbreviations

AML: Acute myeloid leukaemia
BC: Blood culture
BSI: Bloodstream infection
CoNS: Coagulase-negative *Staphylococcus*
CVC: Central venous catheter
ESBL: Extended spectrum β-lactamase
FN: Febrile neutropenia
ICU: Intensive care unit
MIC: Minimum inhibitory concentration
qPCR: quantitative polymerase chain reaction
VRE: Vancomycin-resistant enterococcus
